# Does home neighbourhood supportiveness influence the location more than volume of adolescent’s physical activity? An observational study using global positioning systems

**DOI:** 10.1186/s12966-017-0607-7

**Published:** 2017-11-02

**Authors:** Emma Coombes, Andy Jones, Ashley Cooper, Angie Page

**Affiliations:** 1 Norwich Medical School, University of East Anglia, Norwich, UK; 2 UKCRC Centre for Diet and Activity Research (CEDAR), Institute of Public Health, Cambridge, UK; 3 Centre for Exercise, Nutrition and Health Sciences, University of Bristol, Bristol, UK

**Keywords:** Adolescents, Physical activity, Global positioning systems, Neighbourhood, Environmental supportiveness

## Abstract

**Background:**

Environmental characteristics of home neighbourhoods are hypothesised to be associated with residents’ physical activity levels, yet many studies report only weak or equivocal associations. We theorise that this may be because neighbourhood characteristics influence the location of activity more than the volume. Using a sample of UK adolescents, we examine the role of home neighbourhood supportiveness for physical activity, both in terms of volume of activity undertaken and a measure of proximity to home at which activity takes place.

**Methods:**

Data were analysed from 967 adolescents living in and around the city of Bristol, UK. Each participant wore an accelerometer and a GPS device for 7 days during school term time. These data were integrated into a Geographical Information System containing information on the participants’ home neighbourhoods and measures of environmental supportiveness. We then identified the amount of out-of-school activity of different intensities that adolescents undertook inside their home neighbourhood and examined how this related to home neighbourhood supportiveness.

**Results:**

We found that living in a less supportive neighbourhood did not negatively impact the volume of physical activity that adolescents undertook. Indeed these participants recorded similar amounts of activity (e.g. 20.5 mins per day of moderate activity at weekends) as those in more supportive neighbourhoods (18.6 mins per day). However, the amount of activity adolescents undertook inside their home neighbourhood did differ according to supportiveness; those living in less supportive locations had lower odds of recording activity inside their home neighbourhood. This was observed across all intensities of activity including sedentary, light, moderate, and vigorous.

**Conclusions:**

Our findings suggest that the supportiveness of the neighbourhood around home may have a greater influence on the location of physical activity than the volume undertaken. This finding is at odds with the premise of the socio-ecological models of physical activity that have driven this research field for the last two decades, and has implications for future research, as by simply measuring volumes of activity we may be underestimating the impact of the environment on physical activity behaviours.

## Background

Public health recommendations suggest that young people should undertake at least 60 min of moderate to vigorous physical activity (MVPA) a day, yet the majority of youth do not meet these guidelines [1]. In addition to overall low levels of physical activity, activity declines during childhood and adolescence, with the mean decline across each year estimated to be approximately 4.2% from age five [2], and with girls showing larger decreases than boys [3, 4]. Decline in physical activity during adolescence is observed across the entire week including during school, after school, and at the weekend [5].

Much attention has focused on the role of characteristics of the built environment, both as a reason for the low levels of physical activity observed and also as a potential solution to promoting increased activity [6]. It is widely hypothesized that the built environment is associated with physical activity because it influences opportunities for both recreational physical activity and active travel (see review papers by e.g. [7–9]). However, despite theoretical models suggesting that built environment features should influence activity levels, systematic reviews have reported equivocal relationships and some counterintuitive findings [10]. The lack of clear associations may partly result from the fact that many studies have focused on neighbourhoods close to homes as the area of interest (e.g. [11, 12]). This has been based on the premise that neighbourhood boundaries can be delineated around home locations, and the characteristics of the area within these boundaries are assumed to be a useful predictor of the physical activity levels of study participants. However, a limitation of this approach is that people may undertake a substantial proportion of their physical activity outside the proximal home neighbourhood [13].

Recent work has extended existing neighbourhood-based research by measuring the spatial activity patterns of individuals. Some work has used self-report methods, for example asking participants to delineate on a map the daily space they make use of (e.g. the VERITAS tool [14]) or by using travel diaries to record the places people commonly visit [15], while other studies have used Global Positioning Systems (GPS) to objectively measure the locations individuals visit by tracking them over several days [16].

Such studies have suggested, perhaps unsurprisingly, that much physical activity takes place outside areas that are typically delineated as corresponding to the home neighbourhood. For example, accelerometry and GPS data collected in a sample of adults living in the North West of England demonstrated that activity was typically undertaken more than 800 m from home [13]. That study also found that the distance from home that physical activity was undertaken varied by participant characteristics and context, including gender, home location, area deprivation, and car ownership. For those living in city locations the median distance that physical activity was undertaken from home was 0.73 km, whereas for those in a town it was 1.34 km, and in rural locations it was much further at 3.54 km. Additionally, there is evidence in adolescents that a further factor influencing the distance from home that activity is undertaken is mode of travel to school. One study found that Canadian 13 year-olds roamed further from home and spent more time being active outside their home neighbourhood if they walked or cycled to school versus being driven or taking the bus [17].

A possible reason why a substantial proportion of physical activity is undertaken outside the home neighbourhood is that individuals will travel to built environment features offering opportunities to be physically active. For example, McCormack et al. [18] found that Australian adults regularly travelled outside their home neighbourhood to visit both formal recreation facilities (e.g. leisure centres, health clubs, swimming pools) and informal recreation facilities (e.g. beaches, rivers, parks) for the purpose of undertaking physical activity. They also found that those living in a neighbourhood that contained more recreation facilities were not willing to travel as far to seek facilities for physical activity as those living in areas with fewer recreation opportunities. This suggests, in that sample at least, that living in a home neighbourhood that was more supportive for physical activity encouraged respondents to undertake physical activity closer to home. Similarly, Villanueva et al. [19] found that home neighbourhood supportiveness for physical activity was associated with the distance children roamed from home, with children living in a location that contained more child-friendly destinations (such as shops and greenspaces) roaming less far than those who lived further from popular destinations.

In addition to the availability of destinations close to home influencing the location of physical activity, general measures of area walkability have also been shown to be influential. For example, Rundle et al. [20] tracked the locations that a sample of adults living in New York City visited over one week using GPS devices. They found that those living in less walkable neighbourhoods, which was measured by the density of street intersections within 1 km of home, made use of a smaller proportion of their home neighbourhood compared to those living in highly walkable neighbourhoods. For example, on average the accessed neighbourhood area was smaller than the area of a 1 km street network buffer, and only 39% of participants, who typically resided in more walkable neighbourhoods, made use of an area larger than this.

Findings from studies of mobility raise the question as to whether the equivocal relationships reported in neighbourhood-based studies are due to the misspecification of appropriate environmental exposures due to their definition being based on proximal zones around homes. To address this issue, we used a sample of 967 adolescents living in the South West of England to examine the role of home neighbourhood supportiveness for physical activity, both in terms of volume of activity undertaken and use of the home neighbourhood as a venue in which activity takes place. Adolescents are an important group because independent mobility increases between the ages of 8–13 years [21], resulting in young people having more freedom without parental supervision. Furthermore, longitudinal studies (e.g. [22]) have demonstrated that physical activity habits developed during adolescence often track through to early adulthood. Consequently, a better understanding of how this key age group make use of the built environment for physical activity could help inform intervention design and policy to prevent the declines in activity typically observed during adolescence.

## Methods

In this study we devised an index of the supportiveness for physical activity of the proximal neighbourhood around the residential locations of a sample of adolescents. We then undertook analysis to investigate the extent to which measured supportiveness was associated with both the volume of physical activity undertaken by each participant (measured using accelerometry) as well as the proximity to home that the activity was undertaken (measured by combining the accelerometry with data from a GPS device in order to identify activity undertaken inside a delineated ‘home neighbourhood’ boundary). Details of the sample, data collection, and analysis are provided below.

### Recruitment

This analysis utilised data from the PEAR (Physical Environment and Activity Relationships) project, which examined the environmental determinants of physical activity in adolescents. A sample of 995 Year 9 adolescents (aged 13-15 yrs, mean 13.5 yrs) were recruited. The sample slightly exceeded the target sample size of 900 adolescents, which was determined using a power calculation. The adolescents were recruited from 15 secondary schools in and around the city of Bristol, UK, with 5 schools each selected from areas of low, medium and high deprivation based on the English Index of Multiple Deprivation score of the school location. The English Index of Multiple Deprivation is an area-based measure of material deprivation based on 7 domains including income, employment, education, health, crime, barriers to housing and services, and the living environment [23]. Data collection took place during school term time between November 2012 and March 2014. A University Ethics Committee approved the study, and written informed consent was obtained from a parent or guardian of all participating adolescents.

### Data collection

Participants were asked to wear an accelerometer (Actigraph GT3X+; Actigraph LLC, FL, USA) at the waist for 7 days during waking hours, which recorded acceleration using a sampling frequency of 30 Hz. They also wore a GPS device (Qstarz BT1000XT; Taipei, Taiwan), attached to the same belt as the accelerometer, which recorded their location at 10 s intervals. In addition, participants completed a questionnaire in which amongst other things they were asked to report their full home address.

Data from the accelerometers were downloaded and re-integrated to a 10 s epoch using Actilife software, after which they were integrated with the GPS data using a custom script in STATA 10 (Statcorp, 2009), based on their date and time-stamps. This produced an activity count with a latitude and longitude coordinate attached (where available) for every 10 s epoch. Periods when the accelerometer count was continuously zero for 60 min or more (allowing for up to two minutes of non-zeros per hour) were excluded from analysis, as these were considered to be times when the accelerometer was not worn [24]. The remaining data points were then classified into four intensity categories: sedentary (≤100 counts per minute (CPM)), light (101–2295 CPM), moderate (2296–4011 CPM), or vigorous activity (≥4012 CPM) [25]. Finally, the data were cleaned to remove GPS locations with low location confidence according to the protocol of Schipperijn et al. [26]. This resulted in the removal of just 0.9% of data points.

### Measures

#### Categorisation of environmental supportiveness

In order to provide a comparison with commonly employed neighbourhood definitions, we delineated home neighbourhoods as the area within a 10-min walk (equivalent to 800 m) around the home address of each participant. Home locations were mapped in a Geographical Information System (ArcGIS 10.1; ESRI Inc.) based on the grid-reference of each adolescent’s full home address obtained using the Ordnance Survey Address Layer 2 product [27]. Neighbourhoods around the home locations were delineated based on distances along the road network.

For each home neighbourhood, three metrics to capture potential environmental supportiveness for physical activity were generated using the GIS. These were selected based on evidence from the literature that they may be associated with physical activity in adolescents. The metrics were the availability of greenspaces (hypothesised to support physical activity e.g. [28]), neighbourhood walkability (with more walkable neighbourhoods hypothesised to encourage physical activity through active travel e.g. [29]) and destinations to visit (with more destinations again hypothesised to encourage physical activity e.g. [30]).

#### Greenspace

Data on the presence of greenspaces was obtained from the Ordnance Survey Open Greenspace 2017 dataset [31]. This dataset provides the locations of all publicly accessible greenspaces in Great Britain including public parks, playgrounds and playing fields. The area of these greenspaces within the home neighbourhood was computed using the GIS and this was converted into a percentage value.

#### Walkability

To measure walkability, we computed the permeability of the road network by taking the commonly used metric of density of junctions per kilometre of road in each neighbourhood [32]. The Ordnance Survey Meridian 1:50,000 scale data [33] was obtained for the year 2012 (to correspond with when the PEAR participants were measured) and provided information on the road network. This was used to compute the number of junctions in each neighbourhood, which was then divided by the total length of roads in kilometres, to give the density of junctions.

#### Destinations to visit

Information on the density of places to visit within the home neighbourhood was obtained from the Ordnance Survey Points of Interest dataset [34] for the year 2012, again to correspond with PEAR data collection, and this detailed the location of all commercial premises. We identified destinations that adolescents were likely to visit either alone or with adults. These covered the broad categories of food (e.g. cafes, fast food takeaway outlets, food shops), leisure (e.g. sports facilities, amusement parks, cinemas), and retail (e.g. clothing, general stores, bookstores). The number of these facilities falling within each neighbourhood was then summed and divided by the area of the neighbourhood in kilometres squared to produce a density measure.

The three metrics above were statistically significantly correlated with each other and therefore they were combined into an index to derive an overall supportiveness score for each home neighbourhood, as well as being examined separately. The index was produced by converting each of the three metrics into normalized scores (standard scores) by dividing each value of the variable by the square root of the sum of squares of all the original values. The normalized scores for the three metrics were then summed to provide an overall combined score. To simplify the presentation of findings, this was used to categorise the home neighbourhoods, where those with a score above the median were classified as “more supportive” whilst those below the median were classed as “less supportive”. For the greenspace variable (represented by the percentage area of greenspace in the neighbourhood) the median value was 4.2% (interquartile range 1.5% - 9.3%), for walkability (represented by the density of junctions per km^2^) the median was 4.0 (interquartile range 2.8–5.1), and for destinations to visit (represented by the density of destinations per km^2^) the median was 16.0 (interquartile range 7.2–38.3).

### Outcome measure

The outcome measure was the number of matched GPS-accelerometer data points of each activity intensity recorded within the home neighbourhood. In order to identify whether or not each data point fell inside the home neighbourhood we mapped each participant’s matched GPS and accelerometer data in the GIS and overlaid this with their home neighbourhood boundary. A count was made of the number of data points that fell inside the boundary for each participant. Separate calculations were undertaken for those data points classified as sedentary, light, moderate, and vigorous intensity activity, identified based on the accelerometer cut-off values. As physical activity undertaken travelling to school will be largely related to distance travelled and travel mode, and that undertaken during the school day is unlikely to be influenced by features of the home neighbourhood, we excluded periods where children would be commuting or at school. We therefore fitted models that included time on weekday evenings (4 pm – midnight; a measure of after school activity), and all day during the weekend as their outcomes.

### Statistical modelling

Statistics were generated describing the sample characteristics, including the number of minutes of different intensities of physical activity accrued according to home neighbourhood supportiveness. Unadjusted differences between those living in a less supportive versus a more supportive neighbourhood were tested using either an Independent Samples *T*-test or a Mann-Whitney U test depending on whether the variable being tested followed a normal distribution.

The association between the number of data points recorded in the home neighbourhood and the supportiveness of the neighbourhood was examined by the use of logistic multiple regression models. The outcome measure was the number of data points recorded inside the home neighbourhood of each physical activity intensity, with the total number of data points of the corresponding activity intensity recorded across the study specified as a denominator, which was fitted as an offset. The denominator was required to account for the fact that different numbers of data points of each intensity were recorded between participants. Logistic regression was used instead of fitting a percentage outcome in a linear model as the range of values could not be less than 0 (none of the data points fell in the neighbourhood) or greater than 100 (all of the data points fell in the neighbourhood), whilst a linear model would allow values that were smaller or greater than these boundaries. As the participants were clustered within schools, random coefficients, with adolescents clustered within schools, were estimated to account for non-independence of outcomes associated with school attended.

We first examined partially adjusted associations, whereby accelerometer wear time was added as a potential confounder, to control for the fact that proximity to home at which physical activity was undertaken may be associated with how much the accelerometer had been worn. We then fitted fully adjusted models. In the fully adjusted models, we further adjusted for the area deprivation of the adolescent’s home location, which was computed in the GIS using English Index of Multiple Deprivation data based on Lower Super Output Area data zones [23]. We adjusted for area deprivation because there is evidence from elsewhere that young people from more deprived areas may travel further to visit facilities such as parks [35]. Finally, we adjusted for a measure of daylength, the number of minutes of daylight between 3 pm and sunset, which was obtained from standard look-up tables.

Separate regression models were fitted for sedentary, light, moderate, and vigorous physical activity. Additionally, the results were stratified by gender and weekday evenings versus weekends. Findings are presented by adjusted odds ratios with confidence intervals. Where adjustment took place, covariates were centred around their means, meaning that parameters presented in the results tables are predicted values at the mean of all covariates. All statistical analyses were undertaken in SPSS v22.

## Results

Out of the 2489 adolescents invited to take part, 995 consented, representing 40.0% of the invited sample. The response rate showed some variation by deprivation; based on national cut-off values, it was 36.9% for schools in the lowest deprivation tertile, 48.5% for those in the middle tertile and 40.7% for schools in the highest. Of the 995 adolescents who took part in the study, 5 were excluded from analysis because they did not provide a valid home address, and a further 23 because they did not provide any GPS data. This left a final sample size of 967 adolescents. Of these, there were more adolescents living in urban locations (*n* = 772, 79.8%), than there were from suburban (*n* = 43, 4.5%) or rural (*n* = 152, 15.7%). Similarly, there were more adolescents from schools in areas in the lowest deprivation tertile (*n* = 505, 52.2%), than there were in the middle (*n* = 190, 19.7%) or highest tertile (*n* = 272, 28.1%) of deprivation. In terms of gender, there were slightly fewer boys (*n* = 413, 42.7%) than there were girls. Additionally, slightly fewer boys (47.7%) compared to girls (51.8%) lived in a home neighbourhood classified as more supportive. In terms of physical activity, boys were more active, undertaking 37.2 mins (95% CI 33.6–40.8) of moderate to vigorous physical activity per day at weekends compared to 25.7 mins (95% CI 23.7–27.7) in girls.

We found that participants living in less supportive neighbourhoods undertook similar amounts of physical activity compared to those in more supportive ones (at weekends 20.5 mins versus 18.6 for moderate activity and 11.9 mins versus 10.3 for vigorous activity, Table 1). Further, when considered relative to device wear time, the percentage of time spent in each activity intensity did not differ strongly between the supportiveness groups. We did find however that participants living in less supportive neighbourhoods spent a smaller percentage of their time inside their neighbourhood; 69.8% of total weekday evening and weekend GPS device wear-time versus 72.4% for those living in more supportive neighbourhoods (results not tabulated).

**Table 1:** Summary of daily physical activity undertaken on weekday evenings and weekends by home neighbourhood supportiveness

Physical activity intensity	Mean mins per day (95% CI)			Percentage of device wear time in activity intensity		
	More supportive	Less supportive	Difference^a^	More supportive	Less supportive	Difference^a^
Weekday evenings	*n* = 484	*n* = 483		*n* = 484	*n* = 483	
Sedentary	122.2 (116.6–127.7)	143.3 (137.9–148.8)	+21.1**	69.9%	71.1%	+1.2
Light	39.1 (37.3–41.0)	45.0 (43.1–46.9)	+5.9**	22.4%	22.3%	−0.1
Moderate	8.5 (7.9–9.1)	8.0 (7.4–8.5)	−0.5	4.9%	4.0%	−0.9**
Vigorous	5.0 (4.5–5.6)	5.2 (4.7–5.8)	+0.2	2.9%	2.6%	−0.3
Weekends	*n* = 445	*n* = 438		*n* = 445	*n* = 438	
Sedentary	290.0 (276.3–303.7)	334.2 (321.6–346.9)	+44.2**	69.8%	68.9%	−0.9
Light	96.4 (91.4–101.3)	118.6 (113.5–123.8)	+22.2**	23.2%	24.4%	+1.2
Moderate	18.6 (17.0–20.1)	20.5 (18.9–22.0)	+1.9	4.5%	4.2%	−0.3
Vigorous	10.3 (8.9–11.7)	11.9 (10.4–13.4)	+1.6	2.5%	2.5%	0

^a^Difference between the supportiveness groups; **p ≤ 0.001

Despite adolescents accruing similar amounts of physical activity irrespective of home neighbourhood supportiveness, the number of data points for each physical activity intensity recorded inside the neighbourhood did differ according to supportiveness. Table 2 shows odds ratios with confidence intervals, with both the partially adjusted and fully adjusted associations presented. After full adjustment the magnitude of difference between the neighbourhood supportiveness groups slightly changed, although direction of effect and statistical significance largely remained unaltered.

**Table 2:** Partially and fully adjusted odds ratios^a^, overall and stratified by gender and time period

Physical activity intensity	Partially adjusted values (odds ratio, 95% CI)			Fully adjusted values (odds ratio, 95% CI)		
	More supportive	Less supportive	Difference^b^	More supportive	Less supportive	Difference^b^
Overall	*n* = 484	*n* = 483		*n* = 484	*n* = 483	
Sedentary	2.604 (2.596–2.612)	2.425 (2.416–2.435)	−0.18**	2.581 (2.572–2.589)	2.455 (2.445–2.465)	−0.13**
Light	2.762 (2.753–2.771)	2.581 (2.570–2.593)	−0.18**	2.735 (2.725–2.744)	2.612 (2.601–2.625)	−0.12**
Moderate	2.951 (2.940–2.961)	2.762 (2.748–2.773)	−0.19**	2.930 (2.919–2.941)	2.793 (2.779–2.804)	−0.14**
Vigorous	2.989 (2.979–3.000)	2.798 (2.784–2.809)	−0.19**	2.959 (2.948–2.971)	2.821 (2.807–2.835)	−0.14**
Boys	*n* = 209	*n* = 204		*n* = 2090	*n* = 204	
Sedentary	2.581 (2.568–2.593)	2.445 (2.430–2.457)	−0.14**	2.707 (2.694–2.721)	2.502 (2.487–2.517)	−0.21**
Light	2.627 (2.614–2.641)	2.565 (2.550–2.581)	−0.06**	2.751 (2.736–2.766)	2.641 (2.625–2.656)	−0.11**
Moderate	2.768 (2.753–2.782)	2.737 (2.721–2.757)	−0.03**	2.892 (2.875–2.909)	2.815 (2.798–2.832)	−0.08**
Vigorous	2.798 (2.783–2.813)	2.762 (2.746–2.782)	−0.04**	2.918 (2.901–2.936)	2.832 (2.815–2.852)	−0.09**
Girls	*n* = 275	*n* = 279		*n* = 275	*n* = 279	
Sedentary	2.643 (2.632–2.654)	2.433 (2.421–2.447)	−0.21**	2.479 (2.468–2.491)	2.401 (2.387–2.416)	−0.08**
Light	2.907 (2.894–2.920)	2.633 (2.617–2.649)	−0.27**	2.716 (2.702–2.729)	2.578 (2.563–2.593)	−0.14**
Moderate	3.136 (3.121–3.151)	2.824 (2.804–2.841)	−0.31**	2.951 (2.936–2.966)	2.768 (2.751–2.787)	−0.18**
Vigorous	3.184 (3.169–3.199)	2.872 (2.855–2.889)	−0.31**	2.995 (2.980–3.010)	2.812 (2.793–2.829)	−0.18**
Weekday evenings	*n* = 484	*n* = 483		*n* = 484	*n* = 483	
Sedentary	3.995 (3.973–4.017)	3.785 (3.762–3.811)	−0.21**	3.834 (3.813–3.855)	3.640 (3.615–3.666)	−0.19**
Light	2.499 (2.478–2.521)	2.259 (2.237–2.284)	−0.24**	2.404 (2.383–2.424)	2.179 (2.155–2.203)	−0.23**
Moderate	1.677 (1.647–1.708)	1.640 (1.603–1.679)	−0.04*	1.519 (1.491–1.547)	1.496 (1.462–1.533)	−0.02
Vigorous	1.501 (1.467–1.535)	1.219 (1.186–1.252)	−0.28**	1.330 (1.300–1.360)	1.093 (1.064–1.124)	−0.24**
Weekends	*n* = 445	*n* = 438		*n* = 445	*n* = 438	
Sedentary	2.002 (1.992–2.012)	1.861 (1.848–1.872)	−0.14**	2.096 (2.086–2.106)	1.950 (1.939–1.962)	−0.15**
Light	1.357 (1.346–1.368)	1.242 (1.230–1.255)	−0.12**	1.429 (1.418–1.441)	1.305 (1.292–1.318)	−0.12**
Moderate	0.980 (0.962–0.999)	0.743 (0.726–0.760)	−0.24**	1.059 (1.039–1.079)	0.796 (0.778–0.815)	−0.26**
Vigorous	0.952 (0.929–0.976)	0.633 (0.614–0.652)	−0.32**	1.010 (0.985–1.036)	0.660 (0.640–0.680)	−0.35**

^a^Odds ratio of recording physical activity inside the home neighbourhood relative to outside

^b^Difference between the supportiveness groups; *p ≤ 0.05, and **p ≤ 0.001

Overall, when models were fitted for both time periods and sexes combined, the results in Table 2 demonstrate that those living in less supportive locations recorded fewer data points within their neighbourhood than their counterparts in more supportive locations, indicating that the proportion of their activity undertaken inside their home neighbourhood was lower. This was the case for all intensities of activity including sedentary, light, moderate and vigorous. For example, prior to stratification, the odds ratio of recording a moderate intensity data point inside the neighbourhood was 2.793 for those in less supportive neighbourhoods versus 2.930 for those in more supportive ones. As adolescents spend relatively short amounts of time undertaking moderate activity, this difference equates to just under 2 min of moderate activity per day.

When we stratified our results by gender (Table 2), the results were similar to those for the overall sample, with both boys and girls consistently recording fewer data points inside their home neighbourhood if they lived in a less supportive location. Similarly, when we stratified by weekday evenings versus weekends, findings did not strongly differ, although there was a general tendency for weekend activity to be less likely to be undertaken inside the home neighbourhood compared to weekday evenings. For example, in terms of the sample overall, around half (54.9%, 95% CI 52.7–57.2) of recorded physical activity took place within the home neighbourhood at weekends and around two thirds (66.1%, 95% CI 64.2–68.1) during weekday evenings (results not tabulated).

Lastly, we repeated our analysis with the three supportiveness metrics (greenspace, walkability and destinations) examined separately (Table 3). Our findings did not differ substantially from those for the combined index.

**Table 3:** Fully adjusted odds ratios^a^ stratified according to the supportiveness metrics

Physical activity intensity	Fully adjusted values (odds ratio, 95% CI)		
	More supportive	Less supportive	Difference^b^
Greenspace	*n* = 484	*n* = 483	
Sedentary	2.547 (2.539–2.555)	2.497 (2.487–2.507)	−0.05**
Light	2.694 (2.684–2.703)	2.667 (2.656–2.678)	−0.03**
Moderate	2.881 (2.870–2.891)	2.852 (2.841–2.866)	−0.03**
Vigorous	2.912 (2.902–2.923)	2.889 (2.875–2.901)	−0.02**
Walkability	*n* = 484	*n* = 483	
Sedentary	2.641 (2.631–2.650)	2.389 (2.377–2.399)	−0.25**
Light	2.804 (2.793–2.815)	2.527 (2.517–2.540)	−0.28**
Moderate	2.989 (2.977–3.002)	2.721 (2.707–2.732)	−0.27**
Vigorous	3.013 (3.001–3.026)	2.757 (2.743–2.770)	−0.26**
Destinations	*n* = 484	*n* = 483	
Sedentary	2.689 (2.680–2.698)	2.319 (2.309–2.328)	−0.37**
Light	2.878 (2.868–2.888)	2.430 (2.421–2.442)	−0.45**
Moderate	3.077 (3.066–3.089)	2.604 (2.591–2.614)	−0.47**
Vigorous	3.111 (3.100–3.123)	2.633 (2.620–2.643)	−0.48**

^a^Odds ratio of recording physical activity inside the home neighbourhood relative to outside

^b^Difference between the supportiveness groups; **p ≤ 0.001

## Discussion

We believe this study provides some novel insights into the influence of home neighbourhood supportiveness on physical activity behaviours. Overall, we found that neighbourhood supportiveness did not appear to be associated with the volume of physical activity that adolescents undertook but it was associated with the location.

The results for the overall sample demonstrated that those in less supportive neighbourhoods were less likely to undertake physical activity inside their home neighbourhood. This was observed across all intensities of activity examined. Although there is evidence from a range of studies (e.g. [19, 36, 37]) that boys will roam further from home than girls, we did not find strong differences by gender when we stratified. Lastly, when we stratified by weekday evenings versus weekends, again the associations between physical activity location and home neighbourhood supportiveness remained in the expected direction and statistically significant, although weekend activity was more likely to be outside the neighbourhood.

In terms of the implications of our findings, without long-term longitudinal data it is not possible to know whether any activity displacement has health implications. It might be that growing up in a less supportive neighbourhood does not significantly affect physical activity levels during youth, but it might help to establish positive attitudes to physical activity. For example, adolescents growing up in less supportive neighbourhoods might develop good active travel behaviours by seeking out places to be active away from home. Alternatively, it could be that the need to use the car to reach more distant places is detrimental from both a health perspective and also an environmental sustainability point of view. We suggest future studies of the environmental correlates of physical activity should measure the locations in which physical activity takes place, not just the volume of activity undertaken; by simply measuring volumes of activity we may be underestimating the impact of the environment on physical activity behaviours. More fundamentally our findings suggest that research should move away from simply drawing neighbourhoods around home locations and focusing on this as the area of interest, as this is likely to poorly capture the relevant environments people use for physical activity. For example, in our sample, only around half (54.9%, 95% CI 52.7–57.2) of recorded physical activity took place within the home neighbourhood at weekends and around two thirds (66.1%, 95% CI 64.2–68.1) during weekday evenings.

This study has a number of strengths and weaknesses. Strengths include that the sample incorporated adolescents living in a range of different settings including urban, suburban, and rural which helped promote environmental heterogeneity, although the number of rural residents (*n* = 152, 15.7%) was too small to allow stratification of analyses by urban-rural status. Furthermore, the sample size was larger than other similar studies (e.g. [38–40]) and it consisted of 97.2% of the total number of PEAR participants who provided valid data. Additionally, both environments and behaviours were objectively measured and analysed using reproducible protocols, hence reducing the possibility of misclassification. Further, we selected our environmental measures based on theory and evidence of possible importance in the literature.

In terms of limitations, the results presented here are based on times the GPS was receiving a satellite signal. Many of our participants will have spent long periods of the day indoors, for example while at home, and it is during these periods that GPS units can fail to record location [26]. As a consequence, it is likely that some physical activity measurements that were recorded indoors have been lost, and it is therefore likely that our measure of in-neighbourhood activity underestimates the true volume undertaken. To investigate the potential impact of this, we repeated our analysis using only GPS data recorded when the participants were deemed highly likely outdoors, based on selecting those GPS data points with signal-to-noise ratio values that sum to ≥250 [41]. Notably, our findings (results not presented) did not differ substantially from those presented here. A further limitation is that we deliberately chose a simple measure of environmental supportiveness but an implication of this is that there may be important features of the environment that we did not measure. For example, some studies have suggested that residential density may be an important correlate of volumes of physical activity undertaken [9]. Additionally, the cross-sectional nature of our analysis means that the differences we observed associated with environmental supportiveness may not be casual, and the use of a waist-worn accelerometer meant that physical activity associated with cycling behaviours is likely to be underrepresented, although the prevalence of cycling is low, with just 2% of English adolescents cycling to school in 2013 [42].

## Conclusions

In conclusion, we found that home neighbourhood supportiveness was not associated with the volume of physical activity that adolescents undertook, but did show some association with an indicator of the location relative to home. Longitudinal work is needed to better understand whether growing up in a less supportive neighbourhood has a beneficial impact on physical activity behaviours longer term, through fostering positive behaviours during youth and into adulthood. Much of the research that has been undertaken on the environmental determinants of physical activity has been based around the socio-ecological models of physical activity that have been published [43]. The general view has been that the features of a neighbourhood will influence the volume of activity but individuals, both adults and children, are increasingly mobile. Choosing to travel some distance to use features of attraction far from home is in some respects more compatible with a social-ecological framework than a static view of the world where such high emphasis is placed on the most proximal environments.

## References

[1] Hallal PC, Andersen LB, Bull FC, Guthold R, Haskell W, Ekelund U (2012). Global physical activity levels: surveillance progress, pitfalls and prospects. Lancet.

[2] Cooper AR, Goodman A, Page AS, Sherar L, Esliger D, van Sluijs EMF, Andersen LB, Anderssen S, Cardon G, Davey R, Froberg K, Hallal P, Janz KF, Kordas K, Kreimler S, Pate RR, Puder JJ, Reilly JJ, Salmon J, Sardinha LB, Timperio A, Ekelund U (2015). Objectively measured physical activity and sedentary time in youth: the international Children’s Accelerometry database (ICAD). Int J Behav Nutr Phys Act.

[3] Brodersen NH, Steptoe A, Boniface DR, Wardle J (2007). Trends in physical activity and sedentary behaviour in adolescence: ethnic and socioeconomic differences. Br J Sports Med.

[4] Wickel EE, Belton S (2016). School's out. .. Now what? Objective estimates of afterschool sedentary time and physical activity from childhood to adolescence. J Sci Med Sport.

[5] Harding SK, Page AS, Falconer C, Cooper AR (2015). Longitudinal changes in sedentary time and physical activity during adolescence. Int J Behav Nutr Phys Act.

[6] Transport Research Board. Does the built environment influence physical activity? Examining the evidence. TRB special report no. 282. Washington DC: National Acad Sci; 2005. pp. 1-248.

[7] Davison KK, Lawson CT (2006). Do attributes in the physical environment influence children's physical activity? A review of the literature. Int J Behav Nutr Phys Act.

[8] Biddle SJH, Atkin AJ, Cavill N, Foster C (2011). Correlates of physical activity in youth: a review of quantitative systematic reviews. Int Rev Sport Exerc Psychol.

[9] Ding D, Sallis JF, Kerr J, Lee S, Rosenberg DE (2011). Neighborhood environment and physical activity among youth: a review. Am J Prev Med.

[10] McCormack GR, Shiell A (2011). Search of causality: a systematic review of the relationship between the built environment and physical activity among adults. Int J Behav Nutr Phys Act.

[11] Rosenberg D, Ding D, Sallis JF, Kerr J, Norman GJ, Durant N, Harris SK, Saelens BE (2009). Neighborhood environment walkability scale for youth (NEWS-Y): reliability and relationship with physical activity. Prev Med.

[12] Tucker P, Irwin JD, Gilliland J, He M, Larsen K, Hess P (2009). Environmental influences on physical activity levels in youth. Health Place..

[13] Hillsdon M, Coombes E, Griew P, Jones A (2015). An assessment of the relevance of the home neighbourhood for understanding environmental influences on physical activity: how far from home do people roam?. Int J Behav Nutr Phys Act.

[14] Chaix B, Kestens Y, Perchoux C, Karusisi N, Merlo J, Labadi K (2012). An interactive mapping tool to assess individual mobility patterns in neighborhood studies. Am J Prev Med.

[15] Winters M, Voss C, Ashe MC, Gutteridge K, McKay H, Sims-Gould J (2015). Where do they go and how do they get there? Older adults' travel behaviour in a highly walkable environment. Soc Sci Med.

[16] Kerr J, Duncan S, Schipperjin J (2011). Using global positioning systems in health research: a practical approach to data collection and processing. Am J Prev Med.

[17] Loebach JE, Gilliland JA (2016). Free range kids? Using GPS-derived activity spaces to examine Children’s neighborhood activity and mobility. Environ Behav.

[18] McCormack G, Giles-Corti B, Bulsara M, Pikora TJ (2006). Correlates of distances traveled to use recreational facilities for physical activity behaviors. Int J Behav Nutr Phys Act.

[19] Villanueva K, Giles-Corti B, Bulsara M, McCormack GR, Timperio A, Middleton N, Beesley B, Trapp G (2012). How far do children travel from their homes? Exploring children's activity spaces in their neighbourhood. Health Place..

[20] Rundle AG, Sheehan DM, Quinn JW, Bartley K, Eisenhower D, Bader MMD, Lovasi GS, Neckerman KM (2016). Using GPS data to study neighborhood walkability and physical activity. Am J Prev Med.

[21] Schoeppe S, Duncan MJ, Badland HM, Oliver M, Browne M (2014). Associations between children’s independent mobility and physical activity. BMC Public Health.

[22] Kjønniksen L, Torsheim T, Wold B (2008). Tracking of leisure-time physical activity during adolescence and young adulthood: a 10-year longitudinal study. Int J Behav Nutr Phys Act.

[23] Department for Communities and Local Government. English indices of deprivation. www.gov.uk/government/statistics/english-indices-of-deprivation-2015. Accessed 22 August 2017.

[24] Troiano RP, Berrigan D, Dodd KW, Mâsse LC, Tilert T, McDowell M (2008). Physical activity in the United States measured by accelerometer. Med Sci Sports Exerc.

[25] Evenson KR, Catellier DJ, Gill K, Ondrak KS, McMurray RG (2008). Calibration of two objective measures of physical activity for children. J Sport Sci.

[26] Schipperijn J, Kerr J, Duncan S, Madsen T, Klinker CD, Troelsen J (2014). Dynamic accuracy of GPS receivers for use in health research: a novel method to assess GPS accuracy in real-world settings. Front Public Health.

[27] Ordnance Survey. Address Layer 2: about the product. www.ordnancesurvey.co.uk/business-and-government/help-and-support/products/addresslayer2.html. Accessed 24 April 2017.

[28] Janssen I, Rosu A (2015). Undeveloped green space and free-time physical activity in 11 to 13-year-old children. Int J Behav Nutr Phys Act.

[29] Carlson JA, Saelens BE, Kerr J, Schipperijn J, Conway TL, Frank LD, Chapman JE, Glanz K, Cain KL, Sallis JF (2015). Association between neighborhood walkability and GPS-measured walking, bicycling and vehicle time in adolescents. Health Place.

[30] Carver A, Veitch J, Sahlqvist S, Crawford D, Hume C (2014). Active transport, independent mobility and territorial range among children residing in disadvantaged areas. J Transp Health.

[31] Ordnance Survey. Open Greenspace: about the product. www.ordnancesurvey.co.uk/business-and-government/products/os-open-greenspace.html. Accessed 22 August 2017.

[32] Panter JR, Jones AP, van Sluijs EMF (2008). Environmental determinants of active travel in youth: a review and framework for future research. Int J Behav Nutr Phys Act.

[33] Ordnance Survey. Meridian: about the product. www.ordnancesurvey.co.uk/business-and-government/help-and-support/products/meridian2.html. Accessed 24 April 2017.

[34] Ordnance Survey. Points of Interest: about the product. www.ordnancesurvey.co.uk/business-and-government/products/points-of-interest.html. Accessed 24 April 2017.

[35] Veitch J, Salmon J, Ball K (2008). Children's active free play in local neighbourhoods: a behavioural mapping study. Health Educ Res.

[36] Mackett R, Brown B, Gong Y, Kitazawa K, Paskins J (2007). Children’s independent movement in the local environment. Built Environ.

[37] Page AS, Cooper AR, Griew P, Davis L, Hillsdon M (2009). Independent mobility in relation to weekday and weekend physical activity in children aged 10–11 years: the PEACH project. Int J Behav Nutr Phys Act.

[38] Boruff BJ, Nathan A, Nijënstein S (2012). Using GPS technology to (re)-examine operationaldefinitions of ‘neighbourhood’ in place-based health research. Int J Health Geogr.

[39] Rainham DG, Bates CJ, Blanchard CM, Dummer TJ, Kirk SF, Shearer CL (2012). Spatial classification of youth physical activity patterns. Am J Prev Med.

[40] Carlson JA, Schipperijn J, Kerr J, Saelens BE, Natarajan L, Frank LD, Glanz K, Conway TL, Chapman JE, Cain KL, Sallis JF (2016). Locations of physical activity as assessed by GPS in young adolescents. Pediatr.

[41] Kerr J, Marshall S, Godbole S, Neukam S, Crist K, Wasilenko K, Golshan S, Buchner D (2012). The relationship between outdoor activity and health in older adults using GPS. Int J Environ Res Public Health.

[42] Department for Transport (2016). National Travel Survey: England.

[43] Sallis JF, Cervero RB, Ascher W, Henderson KA, Kraft MK, Kerr J (2006). An ecological approach to creating active living communities. Annu Rev Publ Health.

